# Knowledge and Attitude about Basic Life Support and Emergency Medical Services amongst Healthcare Interns in University Hospitals: A Cross-Sectional Study

**DOI:** 10.1155/2019/9342892

**Published:** 2019-03-03

**Authors:** Shahabe A. Saquib, Hassan M. Al-Harthi, Anas A. Khoshhal, Adel A. Shaher, Abdulsalam B. Al-Shammari, AbdulAhad Khan, Tahani A. Al-Qahtani, Imran Khalid

**Affiliations:** ^1^Assistant Professor, Department of Periodontics and Community Dental Sciences, Research Center for Advanced Materials Sciences, King Khalid University, College of Dentistry, Abha, Saudi Arabia; ^2^Resident, King Khalid University, College of Dentistry, Abha, Saudi Arabia; ^3^Demnstrator, Department of Endodontics, Hail University, Saudi Arabia; ^4^Demnstrator, Department of Oral and Maxillofacial Surgery, Hail University, Saudi Arabia; ^5^Assistant professor, Department of Oral and Maxillofacial Surgery, King Khalid University, College of Dentistry, Abha, Saudi Arabia

## Abstract

**Background:**

Road traffic accident and sudden cardiac arrest are one of the most leading causes of death in KSA. Basic life support (BLS) is lifesaving intervention as a premedical facility. Adequate knowledge and awareness about BLS and CPR are mandatory for healthcare students.

**Objective:**

The objective of the present study is to assess the knowledge, awareness and attitude towards BLS among healthcare interns in different university hospitals across Saudi Arabia.* Materials and Method*s. A total of 865 health interns attending the health colleges (Medicine, Dentistry, Nursing, Pharmacy) in the University Hospitals across KSA participated in the study. A self-explanatory questionnaire, comprising of 15 questions, was designed for the study. All the hypotheses were formulated using two-tailed alternatives against each null hypothesis.

**Result:**

Out of 865 subjects, 698 completed the survey with a response rate of (80.69%). The male to female sex ratio in the entire group of respondents was 1.44:1.00. Mean score about the awareness and knowledge of BLS and other emergency services among the participants was 2.74±1.02 and 4.02±1.56 respectively. Female participants revealed significantly higher awareness score than male (*P*-value<0.05). Medical interns showed higher awareness level compared to rest of all the faculty interns (*P*-value<0.01). There was no significant difference in the attitude of interns among the different faculties. 60 to 70% of interns had recommended to include BLS training in the university curriculum.

**Conclusion:**

Among the participants of the study, overall awareness score was average, whereas the knowledge score was below average. Further, the participants showed a positive attitude toward BLS training. An optimistic decision should be considered on the inclusion of Basic Life Support procedures in the university curriculum.

## 1. Introduction

Cardiac arrest is one of the leading causes of death in the modern world. Providers of basic life support (BLS) can intervene early and prevent associated morbidity/mortality. BLS course has changed significantly over the years to make it more valuable to the general population. BLS is an important component of the cardiopulmonary resuscitation (CPR), which includes adequate maintenance of ventilation and circulation in cases of respiratory or cardiac arrest [1].

Adequate knowledge and awareness about BLS and CPR is a vital issue to ensure that individuals can deliver necessary life-saving measures in cases of emergency [2, 3]. It is expected that health care professionals should have a sound knowledge about BLS as they encounter an emergency situation very often [4]. Health care professionals should be competent and confident enough to resuscitate from the very beginning of their courses. In the USA, BLS training course has been suggested for all health care providers since 1966, [5] particularly to those who are involved in resuscitation [6].

The level of knowledge and attitude of health care professionals are variable as evidenced by several surveys conducted in different parts of the world. The demand for BLS courses is ever increasing in the developed countries. However, in the underdeveloped and developing countries, training is not in routinely practiced. A survey conducted in a hospital setup of Nepal revealed that the medical and paramedical professionals are lacking adequate knowledge of CPR/BLS. Only 9 of a total of 121 participants correctly answered more than 11 questions out of 15 [7]. Another study conducted by Vinej* et al*., evaluating the dental interns in a subpopulation of India, showed that there was an obvious lack of knowledge related to the management of medical emergencies. Data from the study revealed that 39.89% had below average knowledge regarding BLS [8].

In Saudi Arabia, current evidence has shown that health care professionals have low levels of BLS knowledge, but optimistic attitude towards acquiring BLS training. A survey done by Al Mesned* et al*., at Qassim university revealed that health care students and healthcare providers had poor knowledge of BLS, which needs to be improved [9]. Another recent study conducted by Alotaibi et al., concluded that dental students and staff had inadequate knowledge regarding basic life support. However, they had positive attitudes towards acquiring it [10]. A survey conducted among the healthcare students at a Saudi women's University inferred that knowledge and awareness about BLS among the female students was very poor; however, attitudes towards acquiring BLS training were positive [11].

To the best of the author's knowledge, there is no existing literature available regarding the knowledge and attitude among health care professionals regarding BLS, in the central and southern region of Saudi Arabia. Therefore the aim of the present study is to assess the knowledge, awareness and attitude towards BLS among the health care professionals during their internship program.

## 2. Materials and Methods

The present prospective, cross-sectional study was conducted from 12 January 2017 to 17 April 2017. A total of 865 healthcare interns attending the health colleges (Medicine, Dentistry, Nursing, Pharmacy) participated in the study. Health universities were randomly selected from each region of Saudi Arabia (Southern, central, western and northern) for the recruitment of participants. The questionnaires, to evaluate the knowledge of BLS, were prepared and updated according to the recent guidelines laid down by the American Heart Association [2]. A self-explanatory questionnaire comprising of 15 questions was designed to assess and compare the knowledge and attitude of basic life support (BLS) among healthcare interns in the University Hospitals across KSA (the Appendix).

The questionnaire comprised of four main domains: (1) Demography, Age, and Faculty of the participants. (2) Awareness of the participants towards emergency services (four questions). (3) The attitude of the participants towards BLS (four questions). (4) Knowledge of the participants towards BLS (eight questions). A pilot study was performed on 20 interns, equally selected from each faculty, to add more credibility to the questionnaire. After pilot testing the questionnaire on a number of subjects, the surveyors did revisions on the basis of suggestions and requests, that were needed for greater understanding. The questionnaire was prepared in the English language, as all the courses were taught in the same language. Face and content validity were assessed and evaluated by the experts in the field of research. Only Saudi nationals were selected for the survey. All the other nationals were excluded rightaway.

The sample size was determined by a previous research that indicated average BLS knowledge scores of 38–45% among the health faculty students [11]. The minimum required sample size, at a 95% confidence interval, a beta value of 0.20 and power of the study as 80, using a test value of 40% allowing a 5% error margin, was 652.

The study protocol was presented before the Institutional Ethics Committee (IEC) and ethical clearance was obtained. The importance of the study was explained verbally to the participants and written informed consent was obtained before filling the questionnaire. The questionnaire did not contain the name of the participants to maintain the confidentiality of the participants. The questionnaire was randomly distributed among the participants during their hospital hours; meanwhile, the research coordinators were around to answer any queries related to it.

All the hypotheses were formulated using two-tailed alternatives against each null hypothesis (hypothesis of no difference). The entire data was statistically analyzed using the Statistical Package for Social Sciences (SPSS version 21.0, IBM Corporation, USA) for MS Windows.

## 3. Results

**Figure 1:**
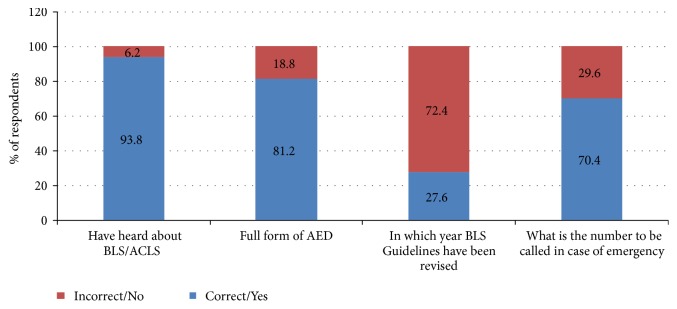
Distribution of Overall Awareness Status.

Out of 865 subjects, 698 completed the survey with a response rate of (80.69%). Among 698 responses received, 56 were incomplete. So they were excluded from the survey. Finally, 642 complete responses were selected for the final statistical analysis. The mean age for the study participants was 24.67±2.56. Of 642 respondents, 379 (59.0%) were males and 263 (41.0%) were females. The male to female sex ratio in the entire group of respondents was 1.44:1.00. The distribution of participants according to gender, age and faculty (college) is shown in detail in Table 1.  Figure 1 represents the distribution of the overall awareness among the respondents. The mean awareness and knowledge scores according to the gender of the respondents are shown in detail in Table 2 with a score range of 0-4 for awareness and 0-7 for knowledge respectively. The distribution of various attitude related responses did not differ significantly between the male and female group of respondents (P-value<0.05 for all).

**Figure 2:**
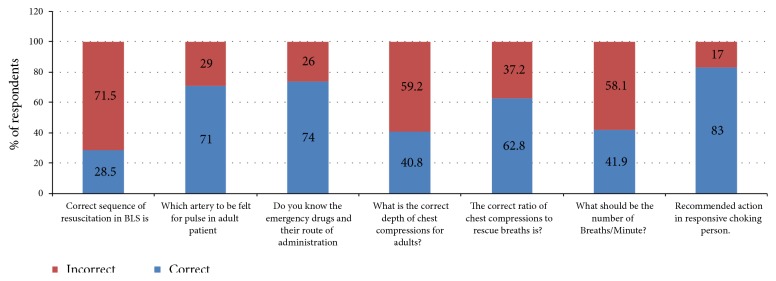
Distribution of Overall Knowledge Status.

Table 3 shows a detailed description of distribution on awareness and knowledge scores according to the college of the respondents. The distribution of mean awareness scores differ significantly across the four faculty group of the respondents (*P*<0.01).  Figure 2 showed that the distribution of mean knowledge score did not differ significantly across the four faculty groups of respondents (*P* <0.05).  Table 4 showed the distribution of various attitude related responses (such as having previous BLS training, the reason for not having BLS training outside college, differs significantly across the four faculty groups of respondents (P-value<0.001 for both). The distribution of self-grading level of knowledge is presented in Table 5 and did not differ significantly across the four faculty groups of respondents (*P*<0.05).

## 4. Discussion

According to the studies and reports, road traffic accidents and sudden cardiac deaths are the major health hazards in Saudi Arabia [12, 13]. In all of the major trauma and cardiac arrest cases, BLS support is required to save life in a prehospital facility. The BLS program is designed to deliver knowledge to a wide range of healthcare professionals about several life-threatening emergencies. It also demonstrates training to provide CPR, in the use of an AED, and to relieve choking in a safe, timely and effective manner to treat those emergencies. It is mandatory for all the health care professionals to have a comprehensive knowledge and skill about BLS. In the present scenario, there is the main problem with skills and obsolete information regarding the BLS [14, 15]. Hence, all healthcare institutes should emphasize in training the students and interns for the BLS. To the best of the author's knowledge, this is the only study evaluating and comparing the knowledge of BLS among the interns of different healthcare facilities across KSA.

In the present study, the mean score about the awareness of BLS and other emergency services among the participants was 2.74 ± 1.02 with a score range of 0-4. These scores are in concordance with the findings of another study in which the mean score was about was 2.94 ± 0.90 with a score range of 0-6 [3]. Although the majority of the subjects from the study group heard about BLS and ACLS, most of the participants were unaware about the recent guidelines for BLS (72.4%). Comparison of awareness from amongst the male and female participants revealed high knowledge scores in the females which was statistically significant. A similar finding was observed in another study from Saudi Arabia and India in which female students achieved significantly higher scores than male students [10, 16]. Statistically, a significant difference was observed while comparing awareness among participants of different faculties. Highest awareness was present in the medical interns (2.94±1.03) followed by nursing (2.74±1.07), pharmacy (2.64±0.91) and lastly the dental interns (2.59±1.11) respectively. The possible reason could be the presence of an emergency course as a part of the medical school curriculum.

The mean score about knowledge of BLS among participants was 4.02±1.56 with a score range of 0-7, as opposed to an Indian study showing a mean score of 1.22 ± 0.91 with a score range of 0-4 [3]. A recent study conducted at women's university in Saudi Arabia revealed that 87.9% of the participants had very poor knowledge scores [17]. Another study conducted on dental students revealed that almost 67% of the participants had poor knowledge score [10]. In our study, comparison of knowledge between male and female participants revealed no significant difference, with score 4.05±1.61 and 3.98±1.56 respectively. Only about 28.5% of the participants answered the correct sequence of Airway, Breathing, and Chest compression. This may be because of the fact that the majority of participants may not be aware of the recent change in the sequence advocated by the American heart association. These findings are comparable to a study done by Akshatha et al., in which only 10% of participants were aware of the new sequence [3]. Also, about 40.8% of the participants provided the correct answer to the depth of chest compression. Around 42% percent of the participants submitted the correct answers regarding the number of breaths per minute. The majority (83%) of the study participants had the knowledge to manage choking individuals. The comparison of knowledge among different faculty was statistically non-significant, with the highest score for medicine (4.13±1.55) and the lowest for pharmacy (3.88±1.51) faculty.

In the present study the majority of the participants showed a positive attitude towards BLS training, which is similar to other studies conducted by Kumar* et al*., Carvalho* et al*., Roshana* et al*., [18–20]. About 91% of the medical and dental interns had previous BLS training sessions, whereas 86% of nursing and 78% and pharmacy interns had previous experience with BLS training. A certain proportion of interns from all the faculties did not receive BLS training in the past. This finding should be explored further by personal interview to evaluate the possible reason for the same. According to a recent study, the knowledge and awareness of the trained subjects were more than the untrained subjects [21]. Reinforcement and refresher courses are necessary for the better cognition. Various authors have recommended that healthcare professionals should have hands-on courses regularly in order to master the skills and refresh knowledge about BLS [22, 23]. Majority of participants among all the faculties showed a positive attitude towards additional BLS training program. These additional sessions will help them to update their knowledge. The study revealed that there is a significant decay of the skill and knowledge about BLS post-6-months of training [15].

When the participants from the various faculties were asked about the inclusion of BLS training program into their course curriculum, 60 to 70% interns replied positively. A recent study conducted by Maha* et al*., showed that internal BLS training performed in colleges showed a better result than external training [17]. Various authors have recommended integrating a BLS training program into the undergraduate curriculum so that the healthcare students will get early exposure to such program [24, 25].

In the current study, time scarcity because of hectic schedule in the university hospitals, was the reason given by the majority of participants for not getting BLS training. About 1/3rd of total participants were ambiguous about the reason for not getting training. Some participants had opted for increased course costs and few of them did not feel the need to get trained for BLS. About 42 to 35% of the participants declared that they have average knowledge about the BLS. A poor score of knowledge was declared by fewer participants (10-15%). The scores differences among different faculties were statistically non-significant. Despite having a good attitude and self-declared average knowledge by the participants, 72% of them were unaware of the recent guidelines for BLS; 71% were unaware of the correct sequence of resuscitation. The similar findings were observed in another study, in which participants stated that they had good knowledge but most of them failed to provide more than 6 correct answers out of 12 questions [3]. Although the study involved interns from all the health care faculties, the study failed to evaluate the skill for BLS among participants. This limitation was because of the fact that the study was questionnaire based. Further studies are recommended to evaluate the actual practical skills of the students and health care workers in KSA.

## 5. Conclusion

Based on the facts received from the study the healthcare interns from the university hospital acquire average awareness and below average knowledge of BLS. Despite having a good attitude toward BLS training, some of the participants have never received BLS training. This issue needs immediate attention to resolve the barriers for not receiving the training. It is recommended that the BLS training program should not only be included in the university curriculum of all healthcare faculties but also be updated at regular intervals.

## Figures and Tables

**Table 1:** Distribution of demographic parameters.

Parameters	Respondents (n)	Respondents %
Gender		
Male	379	59.0
Female	263	41.0
Age Group (years)		
Below 25 years	373	58.1
Above 25 years	269	41.9
Faculty		
Medicine	176	27.4
Dentistry	199	31.0
Nursing	135	21.0
Pharmacy	132	20.6

n=number, %=percentage.

**Table 2:** Distribution of mean awareness and knowledge.

	Male (n=379)		Female (n=263)		*P*-value
Scores	Mean	SD	Mean	SD	
*Awareness Score*	2.65	1.08	2.84	0.98	0.030^*∗*^
*Knowledge Score*	4.05	1.61	3.98	1.53	0.565^NS^

*∗P*-value<0.05 statistically significant, NS-Statistically non-significant.

**Table 3:** Distribution of awareness and knowledge amongst the different faculties.

	Medicine (n=176)		Dental (n=199)		Nursing (n=135)		Pharmacy (n=132)		*P*-value
Scores	Mean	SD	Mean	SD	Mean	SD	Mean	SD	
*Awareness Score*	2.94	1.03	2.59	1.11	2.74	0.91	2.64	1.07	0.008^*∗∗*^
*Knowledge Score*	4.13	1.55	4.02	1.68	3.98	1.52	3.88	1.51	0.441^NS^

*P*-values by ANOVA.* P*-value<0.05 is considered to be statistically significant. *∗∗P*-value<0.01, NS-Statistically non-significant.

**Table 4:** Distribution of attitude in different faculty.

Attitude	Options	Medicine (n=176)		Dental (n=199)		Nursing (n=135)		Pharmacy (n=132)		*P-*value
		n	%	n	%	n	%	n	%	
Have you had previous BLS training?	Yes	159	90.3	182	91.5	116	86	103	78.0	0.001^*∗∗∗*^
	No	17	9.7	17	8.5	19	14	29	22.0	

Do you want more BLS training?	Yes	106	60.2	131	65.8	86	63.7	77	58.3	0.646^NS^
	No	44	25.0	41	20.6	25	18.5	31	23.5	
	Don't know	26	14.8	27	13.6	24	17.8	24	18.2	

Do you think BLS training should be mandatory in the curriculum?	Yes	109	61.9	130	65.3	99	73.3	80	60.6	0.297^NS^
	No	29	16.5	32	16.1	13	9.6	20	15.2	
	Don't know	38	21.6	37	18.6	23	17.0	32	24.2	

If you have had no BLS training outside of college, what was the reason?	it's not important	17	9.7	21	10.6	17	12.6	7	5.3	
	Busy schedule	60	34.1	50	25.1	42	31.1	27	20.5	0.001^*∗∗∗*^
	Not interested	30	17.0	26	13.1	16	11.9	38	28.8	
	Cost of the course	23	13.1	29	14.6	28	20.7	16	12.1	
	No answer	46	26.1	73	36.7	32	23.7	44	33.3	

*P*-values by Chi-Square test. *P*-value<0.05 is considered to be statistically significant. *∗∗∗P*-value<0.001, NS-Statistically non-significant.

**Table 5:** Distribution of self-grading knowledge.

Knowledge	Medicine (n=176)		Dental (n=199)		Nursing (n=135)		Pharmacy (n=132)		P-value
	n	%	n	%	n	%	n	%	
Poor	30	17.0	20	10.1	14	10.4	20	15.2	0.074^NS^
Below Average	37	21.0	63	31.7	37	27.4	33	25.0	
Average	75	42.6	85	42.7	52	38.5	45	34.1	
Good	34	19.3	31	15.6	32	23.7	34	25.8	

*P*-values by Chi-Square test.* P*-value<0.05 is considered to be statistically significant. NS-Statistically non-significant.

## Data Availability

The data used to support the findings of this study are available from the corresponding author upon request.

## Appendix

- Demographic information:
  - Gender: Male   — Female  —
  - College: ……………………………
  - Age: ………years.
- Awareness:
  1. Have you heard about BLS/ACLS?
    - Yes   (b) No
  2. Full form of AED?
      ………………………………
  3. In which year BLS Guidelines have been revised?
      ………………………………
  4. What is the number to be called in case of emergency?
      ………………………………..
- Attitude
  1. Have you had previous BLS training?
    - Yes   (b) No
  2. Do you want more BLS training?
    - Yes
    - No
    - I do not know
  3. Do you think BLS training should be mandatory in the curriculum?
    - Yes
    - No
    - I do not know
  4. If you have had no BLS training outside of college, what was the reason?
    - I think it's not important
    - Busy schedule
    - Not interested
    - Cost of the course
    - No answer
- Knowledge
  1. Correct sequence of resuscitation in BLS is…..
    - Compressions, airway, breathing
    - Airway, breathing, compressions
    - Breathing, airway, compressions
  2. Which artery to be felt for a pulse in an adult patient?
    - Carotid artery
    - Femoral artery
    - Brachial artery
  3. Do you know the emergency drugs and their route of administration?
    - Yes
    - No
  4. What is the correct depth of chest compressions for adults?
    - 5-6 cm
    - 4-5 cm
    - 6-7 cm
    - 2-4 cm
  5. The correct ratio of chest compressions to rescue breaths is
    - 30:2
    - 15:2
    - 30:1
    - 25:2
  6. What should be the number of Breaths/Minute?
    - 10-12 per minute
    - 12-14 per minute
    - 16-18 per minute
    - 20 25 per minute
  7. Recommended action in responsive choking person.
    - Start CPR
    - Attempt abdominal thrusts
    - Perform blind sweeps of the mouth
    - Keep the patient in Trendelenburg position
  8. Grade your knowledge on (BLS)
    - Poor
    - Below average
    - Average
    - Good
